# Protection Against *Helicobacter pylori* Infection in BALB/c Mouse Model by Oral Administration of Multivalent Epitope-Based Vaccine of Cholera Toxin B Subunit-HUUC

**DOI:** 10.3389/fimmu.2018.01003

**Published:** 2018-05-08

**Authors:** Xing Pan, Hong Ke, Xiaojuan Niu, Shan Li, Jun Lv, Longrui Pan

**Affiliations:** ^1^Institute of Infection and Immunity, Taihe Hospital, Hubei University of Medicine, Shiyan, China; ^2^Department of Hemotology, People’s Hospital, Hubei University of Medicine, Shiyan, China; ^3^Department of Pharmacology, Hubei University of Medicine, Shiyan, China

**Keywords:** *Helicobacter pylori*, multivalent vaccine, urease, *H. pylori* adhesion A subunit, cytotoxin-associated antigen, cholera toxin B subunit

## Abstract

Vaccination is an increasingly important alternative approach to control *Helicobacter pylori* infection, since *H. pylori* resistance to previously efficacious antibiotic regimens is increased, and *H. pylori* eradication treatment for upper gastrointestinal diseases is becoming less successful. Fortunately, an efficient oral monovalent *H. pylori* vaccine has been developed. However, compared with monovalent vaccines, multivalent vaccines have the potential to induce more effective and comprehensive protection against *H. pylori* infection. In this study, we designed and produced a multivalent epitope-based vaccine cholera toxin B subunit (CTB)-HUUC with the intramucosal adjuvant CTB and tandem copies of B-cell epitopes (HpaA132-141, UreA183-203, and UreB321-339) and T-cell epitopes (HpaA88-100, UreA27-53, UreB229-251, UreB317-329, UreB373-385, UreB438-452, UreB546-561, CagA149-164, and CagA196-217) from *H. pylori* adhesion A subunit (HpaA), urease A subunit (UreA), urease B subunit (UreB), and cytotoxin-associated antigen (CagA). Serum IgG, stomach, and intestine mucosal sIgA from mice after CTB-HUUC vaccination neutralized *H. pylori* urease activity *in vitro*. CTB-HUUC vaccination promoted *H. pylori*-specific lymphocyte responses and a mixed CD4^+^ T cell immune response as indicated by IFN-γ, interleukin-4, and interleukin-17 production in mice. Both oral prophylactic and therapeutic CTB-HUUC vaccinations reduced gastric urease activity and *H. pylori* infection and protected stomachs in mice. Taken together, CTB-HUUC is a promising potent and safe multivalent vaccine in controlling *H. pylori* infection in BALB/c mouse model.

## Introduction

*Helicobacter pylori* is the most important etiologic factor for upper gastrointestinal diseases including gastritis, peptic ulcer disease, gastric mucosa-associated lymphoid tissue lymphoma, and gastric carcinoma (1, 2). Eradication of *H. pylori* can result in resolution of gastritis and restore a healthy microbiome in the stomach and intestines (3). However, *H. pylori* eradication treatment is becoming less successful for years, and *H. pylori* resistance to previously efficacious antibiotic regimens is increased (4, 5). Therefore, vaccination is an increasingly important alternative approach to control *H. pylori* infection.

The first step for *H. pylori* to colonize in the acidic mammalian stomach is attachment to the gastric mucosa, where adhesins play a critical role in the binding (6, 7). *H. pylori* adhesion A subunit (HpaA) and urease are *H. pylori* adhesins and have been identified as wonderful candidate antigens to develop vaccine against *H. pylori* (8). HpaA has been detected on the surface of *H. pylori*, and it is highly conserved among *H. pylori* strains (9). In addition, genomic studies showed that HpaA conferred with limited sequence homology with other proteins and may act as an *H. pylori*-specific protein (10). Immunization with full length of HpaA or a truncated form of HpaA has shown potent immunogenicity including the ability to protect against *H. pylori* infection in mice (11, 12). *H. pylori* produces large amounts of urease which is composed of two subunits, urease A subunit (UreA) and urease B subunit (UreB) (13). The urease of *H. pylori* can hydrolyze urea to ammonia, thereby neutralizing gastric acid, forming a neutral microenvironment around the bacterium, and facilitating *H. pylori* survival and colonization in human stomach (14). UreA and UreB have been widely used as potential antigens for the development of vaccines against *H. pylori* infection in mice, Mongolian gerbils, nonhuman primates, and humans (15–17). An *H. pylori* vaccine candidate with urease and heat-labile enterotoxin (LT) was assessed in *H. pylori*-free volunteers and had a good immunogenicity profile (18). Cytotoxin-associated antigen (CagA) gene is carried in virulent type I strains of *H. pylori*. CagA^+^ *H. pylori* use a type IV secretion system to transfer CagA into host intestinal epithelial cells, leading to severe gastritis and gastric carcinoma, and CagA was selected as a good vaccine candidate in many studies (19–21). The multivalent *H. pylori* vaccine composed of LT plus vacuolating cytotoxin A (VacA), CagA, and neutrophil-activating protein (NAP) has been found to be immunogenic in *H*. *pylori* negative volunteers (22). Another study reported that the attenuated *Salmonella* vector vaccine, which expressed the fused protein CagA–VacA–UreB can significantly decrease *H. pylori* colonization in mice; and the protection was related to serum IgG and mucosal sIgA antibody responses and specific CD4^+^ T cell T-helper 1 (Th1) type responses (20).

Compared with monovalent vaccines, multivalent vaccines may induce more effective and comprehensive protection against *H. pylori* infection. Guo et al. (23) found that oral immunization with the multivalent vaccine cholera toxin B subunit (CTB)–NAP–UreA–HpaA–HSP60–UreB (CWAE) could induce high levels of antibodies against *H. pylori* antigens, and significantly reduced *H. pylori* colonization in Mongolian gerbils, compared with CTB–UreA–UreB (CTB–UE) or Urease. Flach et al. (12) also found that HpaA_trunc_ (a truncated form of HpaA) is a promising, readily produced, non-toxic antigen for inclusion in a mucosal vaccine against *H. pylori* infection, which may preferably be given together with UreB.

Cholera toxin B subunit is the non-toxic subunit of cholera toxin and can bind cells through GM1 (monosialotetrahexosylganglioside, a glycolipid that is expressed in various cell types such as epithelial cells, neurons, and immune cells) receptors, which then mediates antigen entry into the cell (24). Because of the broad distribution of GM1 ganglioside on various cell types (especially on the luminal surface of intestinal epithelial cells and antigen presenting cells in the gut), CTB has been widely used as a mucosal immunomodulatory agent, and now CTB is also used in the vaccine Dukoral^®^ (a WHO pre-qualified oral cholera vaccine) (25).

The above findings suggested that HpaA, UreA, UreB, and CagA are excellent and promising antigens for vaccine against *H. pylori*. Furthermore, a recent study shows that a multivalent vaccine, which targeted multiple adhesions (urease, Lpp20, HpaA, and CagL) in adherence of *H. pylori* to the gastric mucosa significantly decreased *H. pylori* colonization compared with immunization with urease only, indicated that adhesions which are on the surface of *H. pylori* may be a promising candidate vaccine against *H. pylori* infection (26). The results also suggest that multivalent vaccination may provide better protection than monovalent vaccination. Given the established association of CagA with gastric cancer, a vaccine aimed at preventing this disease should contain CagA (21). In addition, CTB is a safe and efficient mucosal adjuvant and has been exploited in cholera prevention and mucosal vaccine development for decades (25). Therefore, in this study, we formulated and produced a multivalent epitope-based vaccine CTB-HUUC based on three *H. pylori* adhesions (HpaA, UreA, and UreB), one key *H. pylori* virulence factor CagA, and a non-toxic mucosal adjuvant CTB. We evaluated its immunogenicity, immunoreactivity, specificity, prophylactic, and therapeutic efficacy in BALB/c mouse model.

## Materials and Methods

### Animals and Bacteria

Specific pathogen-free (SPF) BALB/c mice, female, 5–6 weeks of age, 14 ± 2 g, were purchased from the Experimental Animal Center of Hubei University of Medicine. This study was approved by the Animal Ethical and Experimental Committee of Hubei University of Medicine.

The mouse-adapted *H. pylori* strain SS1 was obtained from the National Center for Disease Control and Prevention. *H. pylori* was cultured on Columbia blood agar plates enriched with 10% defibrinated horse blood, polymyxin B (161.5 µg/mL), vancomycin (10 µg/mL), trimethoprim (5 µg/mL), and amphotericin B (2.5 µg/mL) under microaerobic conditions (5% O_2_, 10% CO_2_, and 85% N_2_) at 37°C for 3–5 days.

*Helicobacter pylori* lysate preparation: *H. pylori* SS1 were harvested from the plates and suspended in 0.01 M PBS. The suspension mixture was then pulse sonicated (Sonics, USA) for 5 min at 20% capacity while kept in an ice bath. *H. pylori* lysate was snap frozen in liquid nitrogen and kept at −80°C until use.

### Vaccine Design and Production

**Figure 1:**
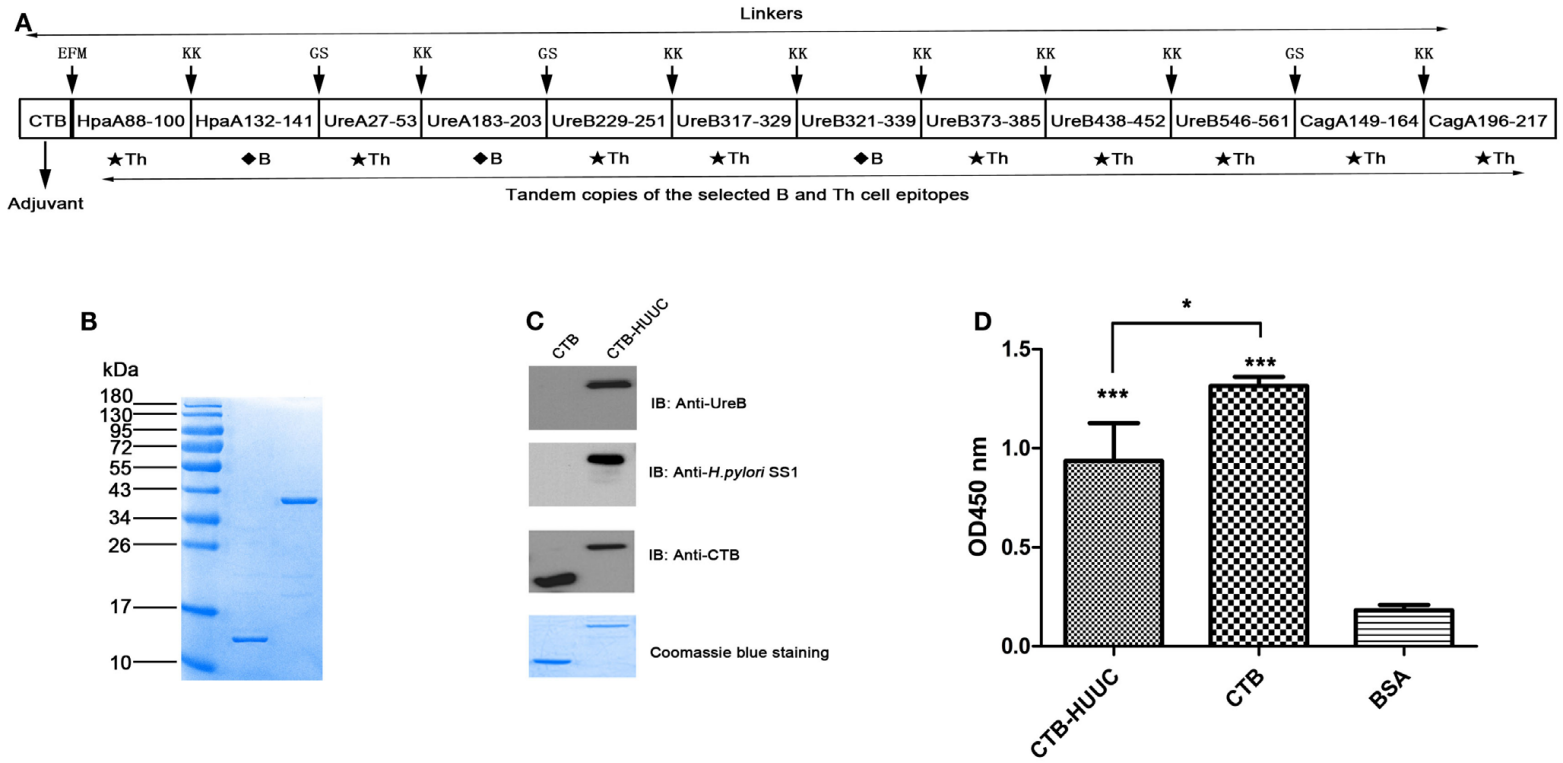
Construction, purification, and verification of cholera toxin B subunit (CTB)-HUUC. **(A)** Composition of the CTB-HUUC construct. **(B)** Visualization of the purified CTB and CTB-HUUC peptides. CTB and CTB-HUUC peptides purified from *Escherichia coli* BL21(DE3) transformed with pET28a(+)/*ctB-huuc*, pET28a(+)/*ctB* were resolved in 12% SDS-PAGE gel and stained with Coomassie Blue. **(C)** Identity verification of CTB and CTB-HUUC using Western blotting. CTB and CTB-HUUC peptides resolved in 12% SDS-PAGE gels were probed with rabbit anti-*Helicobacter pylori* polyclonal antibody, rabbit anti-urease B subunit monoclonal antibody, or mouse anti-CTB monoclonal antibody. **(D)** The adjuvanticity of CTB-HUUC peptide was evaluated using GM1-ELISA. ***p < 0.001, compared with the BSA group, *p < 0.05, compared between the CTB and CTB-HUUC groups.

Four key candidate antigens of *H. pylori* (HpaA, UreA, UreB, and CagA) were selected to construct the multivalent epitope-based vaccine CTB-HUUC. HpaA, UreA, UreB, and CagA sequences were screened for B-cell epitopes and CD4^+^ T-cell epitopes using online B-cell epitope prediction tools (IEDB Analysis Resource^1^) and T-cell epitope prediction tools (IEDB Analysis Resource^2^). Three B-cell epitopes (HpaA132-141, UreA183-203, and UreB321-339) and nine CD4^+^ T-cell epitopes (HpaA88-100, UreA27-53, UreB229-251, UreB317-329, UreB373-385, UreB438-452, UreB546-561, CagA149-164, and CagA196-217) were selected to construct the CTB-HUUC vaccine. GS and KK were selected to link the epitopes in the sequence. Additional sequences of mucosal adjuvant CTB was added to the N-terminus of HUUC. The structure diagram of CTB-HUUC is shown in Figure 1A and Table S2 in Supplementary Material.

The DNA sequences of CTB-HUUC and other vaccines were cloned into respective vectors as the following, pET28a(+)/*ctB-huuc*, pET28a(+)/*ctB*, pSUMO/*hpaA*, pSUMO/*ureA*, pET28a(+)/*ureB*, or pET28a(+)/*cagA* see Table S1 in Supplementary Material. All vaccine proteins CTB-HUUC, CTB, HpaA, UreA, UreB, and CagA were purified by Ni^2+^-charged column chromatography and gel filtration chromatography (GE Healthcare, USA) from *Escherichia coli* BL21(DE3) transformed with the respective recombinant vectors. After purification, the samples were dialyzed against 0.2 M sodium hydrogen carbonate buffer and snap frozen in liquid nitrogen and kept at −80°C until use. The purity of the proteins was assessed by 12% SDS-PAGE.

### Western Blotting

Purified CTB-HUUC and CTB as well as *H. pylori* antigens (HpaA, UreA, UreB, and CagA) were resolved in 12% SDS-PAGE gels and transferred onto polyvinylidene difluoride membrane (PVDF, Millipore, USA). These proteins were probed using primary antibodies including rabbit anti-*H. pylori* polyclonal antibody (prepared by our laboratory), mouse anti-*H. pylori* urease B monoclonal antibody (Sigma, USA), mouse anti-CTB monoclonal antibody (prepared by our laboratory) (27), mouse anti-CTB-HUUC serum, mouse anti-CTB serum, or mouse normal serum. HRP-conjugated goat anti-rabbit IgG (Cell Signaling Technology, USA) or HRP-conjugated goat anti-mouse IgG (Cell Signaling Technology, USA) was used as the secondary antibodies. The results were visualized with ECL chemiluminescence reagents (Millipore, USA).

Mouse anti-CTB-HUUC serum, mouse anti-CTB serum, and mouse normal serum preparation: SPF BALB/c mice were randomized into three groups (*n* = 3 mice, female) and were, respectively, administered orally with 200 µg of antigen (CTB-HUUC, CTB or PBS) in 0.2 M sodium hydrogen carbonate buffer (200 µL) for four times at 1-week interval (at first, second, third, and fourth week), serum samples were collected from the submandibular vein in each groups at second, third, fourth, fifth, and seventh week.

### GM1-ELISA

ELISA plates (Wuhan Fine Biotech Co., Ltd., China) were coated with GM1 ganglioside or BSA (1 μg/well) at 4°C for 12 h. After washing with PBST buffer, ELISA plates were incubated with 5% skim milk at 37°C for 1 h. The CTB-HUUC, CTB, or BSA proteins (10 μg/well) were then added to ELISA plates and incubated at 37°C for 2 h. After that, a proper dilution of anti-CTB monoclonal antibody was added to the plates and incubated at 37°C for 1 h. After washing with PBST buffer, HRP-conjugated goat anti-mouse IgG was added to the plate and incubated at 37°C for 1 h. Substrate tetramethylbenzidine (TMB) (Sigma, USA) was then added and incubated at 37°C for 15 min. The absorbance was measured at 450 nm using a microplate reader (PerkinElmer, USA).

### Prophylactic and Therapeutic Vaccination

Prophylactic vaccination (Figure 5A): mice were randomly divided into three groups (*n* = 11 mice, female) and were respectively vaccinated intragastrically with 200 µg of antigen (CTB-HUUC, CTB, or PBS) in 0.2 M sodium hydrogen carbonate buffer (200 µL) for four times at 1-week interval (at sixth, seventh, eighth, and ninth week). Two weeks after the final immunization (at 11th week), mice were infected with *H. pylori* SS1 [10^9^ colony-forming units (CFU) once, 50 µL, four times at 1-day interval]. Mice were sacrificed at 14th week.

Therapeutic vaccination (Figure 6A): mice were randomized into three groups (*n* = 11 mice, female), and infected with *H. pylori* SS1 (10^9^ CFU once, 50 µL, four times at 1-day interval) at sixth week. From the 11th to 14th week, *H. pylori* SS1 infected mice were, respectively, vaccinated intragastrically with 200 µg of antigen (CTB-HUUC, CTB or PBS) in 0.2 M sodium hydrogen carbonate buffer (200 µL) for four times at 1-week interval. Two weeks after the final immunization (at 16th week), mice were sacrificed and examined.

### Determination of Specific IgG or IgA Levels in Serum, Stomach, and Intestine Mucosa

ELISA plates were coated with CTB-HUUC (2 μg/well), *H. pylori* urease (Creative Enzymes, USA) (2 μg/well), CTB (2 μg/well), HpaA (2 μg/well), UreA (2 μg/well), UreB (2 μg/well), or CagA (2 μg/well) at 4°C overnight and were blocked with 5% BSA. Each sample was added to the antigens coated plate, and HRP-conjugated goat anti-mouse IgG or IgA antibodies was added and incubated at 37°C for 1 h. Finally, TMB was added and incubated at 37°C for 10 min. The reaction was then stopped with 2 M H_2_SO_4_. The absorbance was measured at 450 nm using a microplate reader.

Sample preparation: 2 weeks after the final immunization, the serum samples were collected from the submandibular vein and diluted 1:1,000 before assay, after that, mice were sacrificed. To determine specific stomach and intestine mucosal secretory IgA (sIgA) levels, one-fourth of stomach tissue or half of intestine tissue (dodecadactylon) was homogenized in 1 mL PBS containing a protease inhibitor mixture (Roche, Germany) and 0.05 M ethylenediaminetetraacetic acid. The supernatant was collected and diluted 1:10 for assay.

### Determination of the Antibody Levels According to Neutralization of *H. pylori* Urease Activities

Serum, stomach mucus, and intestine mucus were collected from mice immunized with CTB-HUUC, CTB, or PBS. Serum IgG antibodies [anti-(CTB-HUUC) or anti-CTB] were purified by protein G column chromatography (GE Healthcare, USA). Serum, stomach mucus, and intestine mucus, or purified IgG antibodies were incubated the purified *H. pylori* urease (Creative Enzymes, USA) in 100 µL PBS (50 mM, pH 6.8) containing 0.5 M urea, 0.1 mM dithiothreitol (DTT) overnight at 4°C, and 0.02% phenol red was added to each well. The absorbance was measured at 550 nm using a microplate reader. Percentage inhibition = [(activity without antibodies − activity with antibodies)/(activity without antibodies)] × 100%.

### Determination of Specific T Lymphocyte Response and IFN-γ, IL-4, and IL-17 Production

Lymphocyte suspensions were prepared from the mice spleen and cultured with the antigen Concanavalin A (ConA) (Sigma, USA), *H. pylori* lysates (prepared by our laboratory), *H. pylori* urease, or CTB in RPMI-1640 for 72 h. After that, 10 µL CCK-8 solution (Cell Counting Kit-8) (Beijing Solarbio Science & Technology Co., Ltd., China) was added into plates and incubated for 4 h. The absorbance was measured at 450 nm using a microplate reader. The results are expressed as SI. SI = Stimulated cultures (OD 450 nm)/Negative control cultures (OD 450 nm).

To measure interferon gamma (IFN-γ), interleukin-4 (IL-4), and interleukin-17 (IL-17) production, lymphocytes (2 × 10^5^ cells/well) were cultured with the *H. pylori* lysates in RPMI-1640 for 96 h. The culture supernatants were collected for determination of IFN-γ, IL-4, and IL-17 levels using ELISA Kits (Thermo Fisher Scientific, USA).

### Examination of *H. pylori* Colonization in Stomachs

To examine the *H. pylori* colonization in the stomachs, mice were sacrificed after the final prophylactic vaccination (at 14th week) or therapeutic vaccination (at 16th week). One-fourth of stomach tissue samples were homogenized in 1 mL PBS and prepared for a serial 10-fold dilutions. These diluted homogenates were spread on the *H. pylori* selective plates. After culture for 3–5 days, bacterium colonies were counted, and the number of CFU per stomach was calculated.

The levels of *H. pylori* colonization in mice stomachs were also evaluated by rapid urease test. Briefly, one-fourth of stomach tissue samples were immediately put in 500 µL of sodium phosphate buffer containing 0.5 M urea, 0.02% phenol red and 0.1 mM DTT, incubated at 37°C for 3 h. The absorbance was measured at 550 nm using a microplate reader.

### Histological Analysis

One-fourth of stomach tissue samples were fixed with formalin, embedded in paraffin and cut to 4 µm slices. Hematoxylin and eosin staining was then performed according to the standard procedure.

### Statistical Analysis

All independent experiments carried out in this study and indicated in the figure legends were biological replicates. The statistical analysis was performed using SPSS 17.0 software. One-way ANOVA was used to compare the differences between groups. *P* < 0.05 was considered as statistically significant.

## Results

### Production and Antigenic Characterization of CTB-HUUC Peptide

To obtain pure peptides as vaccine, we transformed *E. coli* BL21(DE3) with the recombinant vector pET28a(+)/*ctB-huuc* (Figure 1A), pET28a(+)/*ctB*, pSUMO/*hpaA*, pSUMO/*ureA*, pET28a(+)/*ureB*, or pET28a(+)/*cagA* and purified CTB-HUUC, CTB, HpaA, UreA, UreB, and CagA peptides. We analyzed the purity and identity of these peptides with SDS-PAGE and Western blotting. The results showed that the purity of CTB-HUUC was 97.6% and CTB was 98.3% (Figure 1B). CTB-HUUC protein was recognized by rabbit anti-*H. pylori* polyclonal antibody and mouse anti-*H. pylori* UreB monoclonal antibody (Figure 1C). We analyzed the adjuvanticity of CTB component in CTB-HUUC Western blotting and GM1-ELISA and found that CTB-HUUC can bind GM1 *in vitro* (Figures 1C,D).

**Figure 2:**
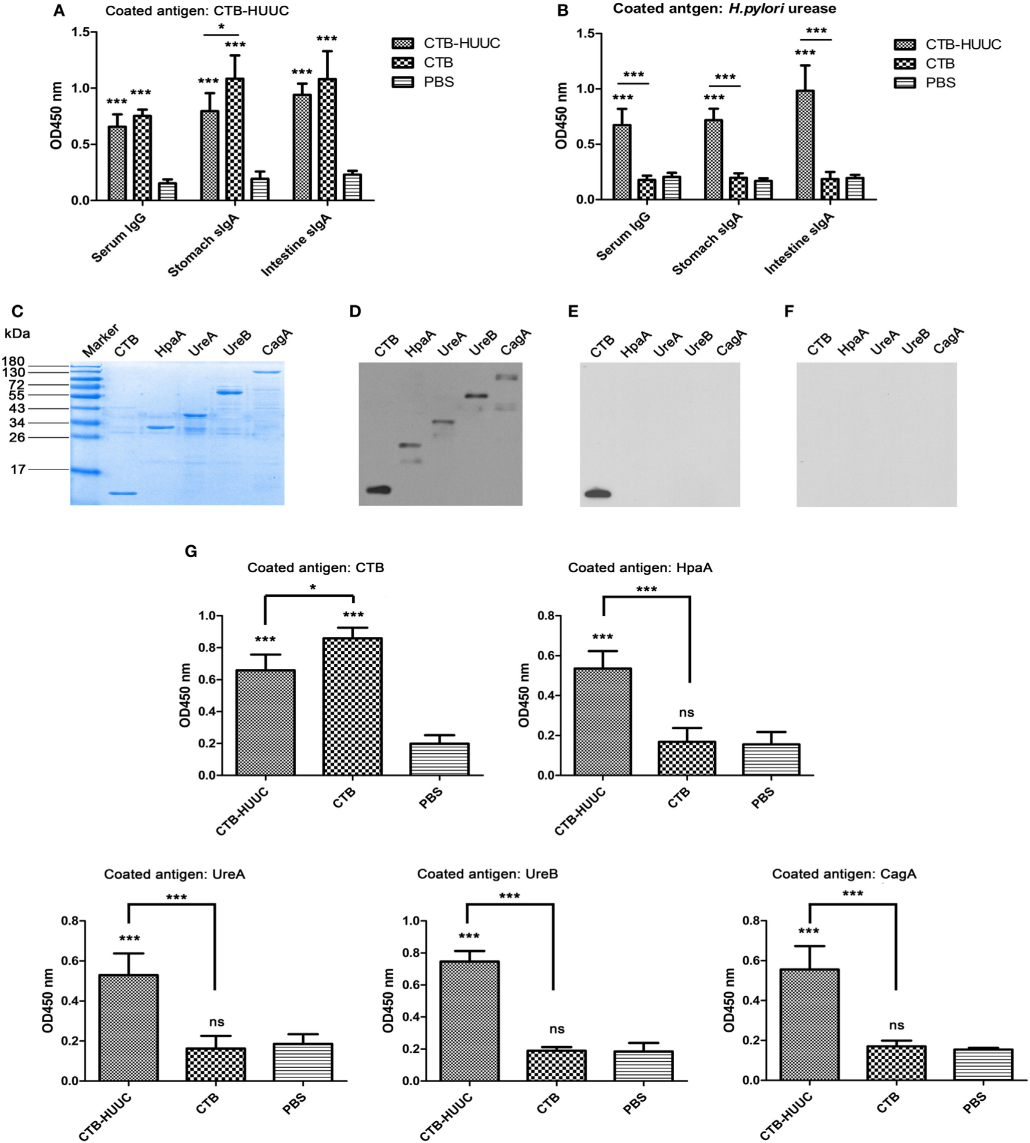
Specific IgG or IgA levels in immunized mice serum, gastric mucus, and intestinal mucus. Serum, stomach, and intestine tissue samples were collected from mice immunized with cholera toxin B subunit (CTB)-HUUC, CTB, or PBS. The levels of serum IgG, and stomach and intestine mucosal IgA against CTB-HUUC **(A)** or *Helicobacter pylori* urease **(B)** were determined by ELISA. ***p < 0.001, compared with the PBS group, *p < 0.05, compared between the CTB-HUUC and CTB groups. **(C)** Visualization of the adjuvant CTB and the *H. pylori* antigens [*H. pylori* adhesion A subunit (HpaA), urease A subunit (UreA), urease B subunit (UreB), and cytotoxin-associated antigen (CagA)] purified from *Escherichia coli* BL21(DE3) transformed with pET28a(+)/*ctB*, pSUMO/*hpaA*, pSUMO/*ureA*, pET28a(+)/*ureB*, or pET28a(+)/*cagA*. These purified peptides were resolved in 12% SDS-PAGE gel and stained with Coomassie Blue. The resolved peptides in 12% SDS-PAGE gels were also probed by antiserum collected from mice immunized with CTB-HUUC **(D)**, CTB **(E)**, or PBS **(F)**. **(G)** The specificity of serum from mice immunized with CTB-HUUC was analyzed by ELISA. ***p < 0.001 and *p < 0.05 compared with the PBS group or between the CTB-HUUC and the CTB groups.

We further evaluated specific IgG or IgA levels in the serum, and gastric mucus and intestinal mucus in mice immunized with CTB-HUUC, CTB, or PBS using ELISA. The results showed that oral immunization with CTB-HUUC significantly increased levels of specific serum IgG, stomach mucosal secretory IgA (sIgA) and intestine mucosal sIgA antibodies compared with the PBS group (*P* < 0.001) (Figures 2A,B). We also examined the specificity of serum from mice immunized with CTB-HUUC using purified CTB, HpaA, UreA, UreB, and CagA peptides and Western blotting (Figures 2C–F) and ELISA (Figure 2G). The results found that serum from mice immunized with CTB-HUUC recognized CTB, HpaA, UreA, UreB, and CagA peptides, serum from mice immunized with CTB only recognized CTB, and serum from mice immunized with PBS did not recognize any of CTB, HpaA, UreA, UreB, and CagA (Figures 2D–G). These results indicated that CTB, HpaA, UreA, UreB, and CagA in CTB-HUUC have good immunogenicity and immunoreactivity, and CTB-HUUC is a multivalent vaccine.

### CTB-HUUC Vaccination Generated Serum IgG, Stomach, and Intestine Mucosal sIgA Inhibited *H. pylori* Urease Activity

**Figure 3:**
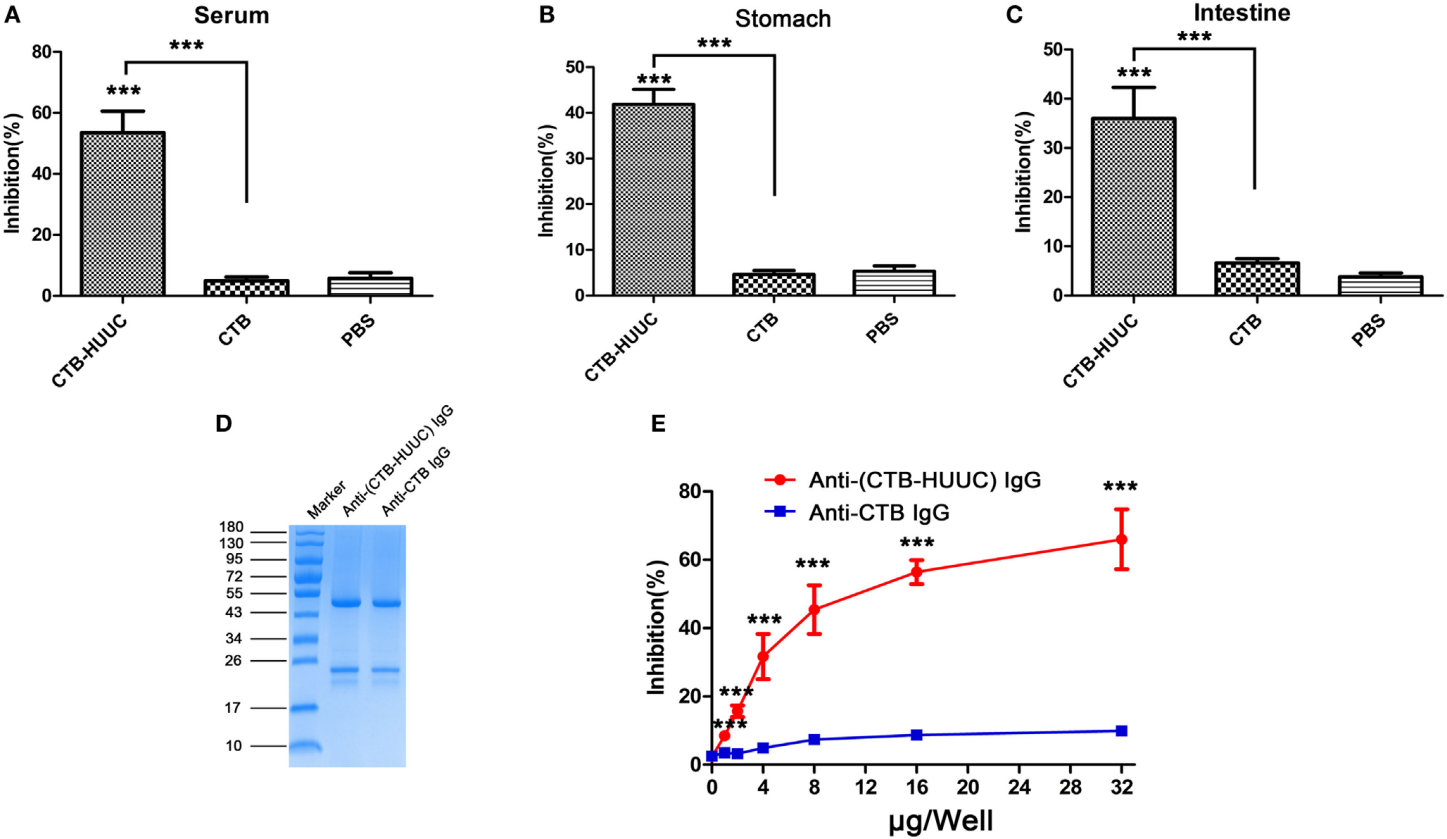
Inhibition of *Helicobacter pylori* urease activity by IgG or IgA from mice immunized with cholera toxin B subunit (CTB)-HUUC, CTB, or PBS. Serum **(A)**, stomach mucus **(B)**, and intestine mucus **(C)** from mice immunized with CTB-HUUC, CTB, or PBS were incubated with *H. pylori* urease. The *H. pylori* urease activity determined using neutralization assay. ***p < 0.001, compared with the PBS group or between the CTB-HUUC and the CTB groups. **(D)** Visualization of IgG purified from serum of mice immunized by CTB-HUUC or CTB. The purified IgG was resolved in 12% SDS-PAGE gel and stained with Coomassie Blue. **(E)** Neutralization of urease activity by IgG purified from serum of mice immunized by CTB-HUUC or CTB. *H. pylori* urease was pre-incubated with the purified serum IgG (0, 1, 2, 4, 8, 16, and 32 μg/well). The optical density of the mixture was determined at 550 nm. The data are expressed as percentage inhibition. ***p < 0.001, compared with the PBS group or between the CTB-HUUC and the CTB groups.

To further evaluate the effects of CTB-HUUC induced antibodies on *H. pylori* urease activity, we performed a urease neutralization assay. The results showed that only serum or supernatants of homogenized stomachs or intestines from mice immunized with CTB-HUUC but not from mice immunized with CTB or PBS inhibited *H. pylori* urease activity (Figures 3A–C). After that, mouse anti-(CTB-HUUC) IgG and mouse anti-CTB IgG in the antiserum were purified by protein G column chromatography (Figure 3D). Using purified antibodies, we found that mouse anti-(CTB-HUUC) IgG inhibited the urease activity in a dose-dependent manner, while mouse anti-CTB IgG did not inhibit the urease activity (Figure 3E).

### CTB-HUUC Vaccination Promoted *H. pylori*-Specific Lymphocyte Responses and IFN-γ, IL-4, and IL-17 Production in Mice

**Figure 4:**
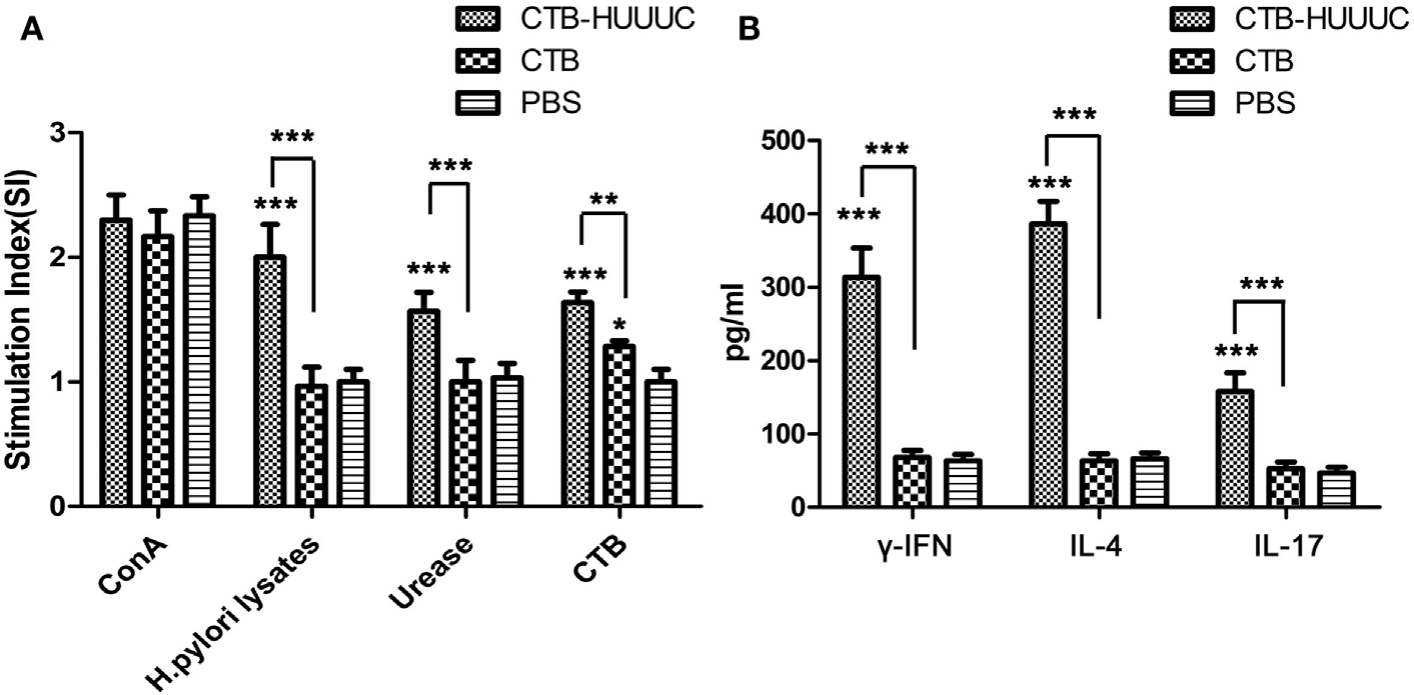
*Helicobacter pylori*-specific lymphocyte responses and IFN-γ, interleukin-4 (IL-4), and interleukin-17 (IL-17) production in mice immunized with cholera toxin B subunit (CTB)-HUUC or CTB. **(A)** Assessment on proliferation of specific lymphocytes in mice after immunization with CTB-HUUC, CTB, or PBS. Splenic lymphocytes from mice immunized with CTB-HUUC, CTB, or PBS were stimulated with Concanavalin A (ConA), *H. pylori* lysates, *H. pylori* urease, or CTB. Cell proliferation was determined using CCK-8 assay. The results were expressed as stimulation indices (SI) which represents the ratio between the proliferation rates of cells stimulated with antigens and those with the vehicle control. *p < 0.05, **p < 0.01, and ***p < 0.001, compared with the PBS group or between the CTB-HUUC and CTB groups. **(B)** The concentrations of IFN-γ, IL-4, and IL-17 in the supernatants of lymphocytes cultures. Splenic lymphocytes from mice immunized with CTB-HUUC, CTB, or PBS were stimulated with *H. pylori* lysates and the supernatants were collected for determination of IFN-γ, IL-4, and IL-17 concentrations by ELISA. ***p < 0.001, compared with the PBS group or between the CTB-HUUC and CTB groups.

To evaluate the potential capacity of CTB-HUUC to stimulate lymphocyte-specific responses for *H. pylori*, we isolated splenic lymphocytes from mice immunized with CTB-HUUC, CTB, or PBS, stimulated them with ConA (a lectin extracted from the jack-bean and well known for its ability to stimulate T cells proliferation), *H. pylori* urease, *H. pylori* lysates, or CTB and performed a cell proliferation assay (CCK-8 assay). The results were expressed as SI which represents the ratio between the proliferation rates of cells stimulated with antigens and those with the vehicle control. The data showed that ConA significantly increased proliferation of splenic lymphocytes from mice orally immunized with CTB-HUUC, CTB, or PBS, as expected (Figure 4A). Both *H. pylori* lysates and *H. pylori* urease significantly increased proliferation of splenic lymphocytes from mice orally immunized with CTB-HUUC, but not those with CTB or PBS (Figure 4A). CTB also increased proliferation of splenic lymphocytes from mice immunized with CTB-HUUC or CTB, compared with PBS vaccination. These results indicated that CTB-HUUC induced specific lymphocyte responses against CTB and *H. pylori*.

We further determined the concentrations of IFN-γ, IL-4, and IL-17 in the supernatants of cultured and *H. pylori* lysate stimulated lymphocytes from mice immunized with CTB-HUUC, CTB, or PBS using ELISA. The results showed that *H. pylori* lysate significantly induced high levels of IFN-γ, IL-4, and IL-17 in splenic lymphocytes from mice orally immunized with CTB-HUUC, but not those with CTB or PBS (Figure 4B).

### Prophylactic or Therapeutic CTB-HUUC Vaccination Reduced Gastric *H. pylori* Infection and Protected Stomachs in BABL/c Mouse Model

**Figure 5:**
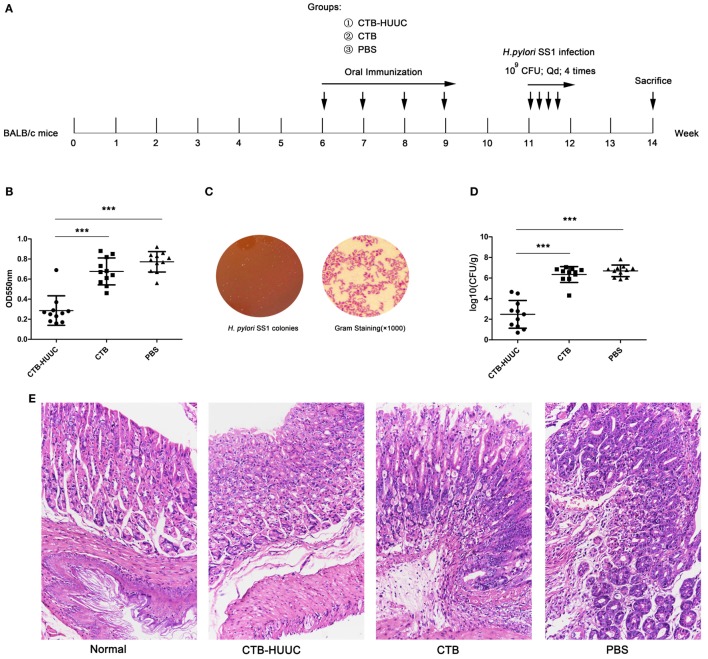
The effects of prophylactic vaccination with cholera toxin B subunit (CTB)-HUUC or CTB on *Helicobacter pylori* infection. **(A)** The prophylactic vaccination procedure. **(B)** The relative levels of urease activity in the stomach of mice infected by *H. pylori* after prophylactic oral immunization. ***p < 0.001, compared with the PBS group or between the CTB-HUUC and CTB groups. **(C)** Representative view of the *H. pylori* culture for colony formation assay. **(D)** The colony-forming units (CFUs) of *H. pylori* infection in the stomach of mice after prophylactic oral immunization. *H. pylori* infection was determined using quantitative culture. Abbreviation: CFU, colony-forming unit. ***p < 0.001, compared with the PBS group or between the CTB-HUUC and CTB groups. **(E)** Gastric histological examination of mice infected by *H. pylori* after prophylactic oral immunization. The gastric tissue samples stained with hematoxylin and eosin were examined under a microscope (200×).

Since the CTB-HUUC antigen showed good immunogenicity, immunoreactivity, and specificity, we further investigated whether oral vaccination with CTB-HUUC reduces the bacterium load in the stomachs of BABL/c mouse model infected with *H. pylori* SS1 and shows better prophylactic or therapeutic effect than CTB or PBS. The results found that prophylactic CTB-HUUC vaccination significantly decreased urease activity and reduced the bacterium load in the stomachs, compared with CTB or PBS vaccination (*P* < 0.001) (Figures 5B–D), and prophylactic CTB-HUUC vaccination also provided a better protection for stomachs than CTB did according to the gastric histological examination (Figure 5E).

**Figure 6:**
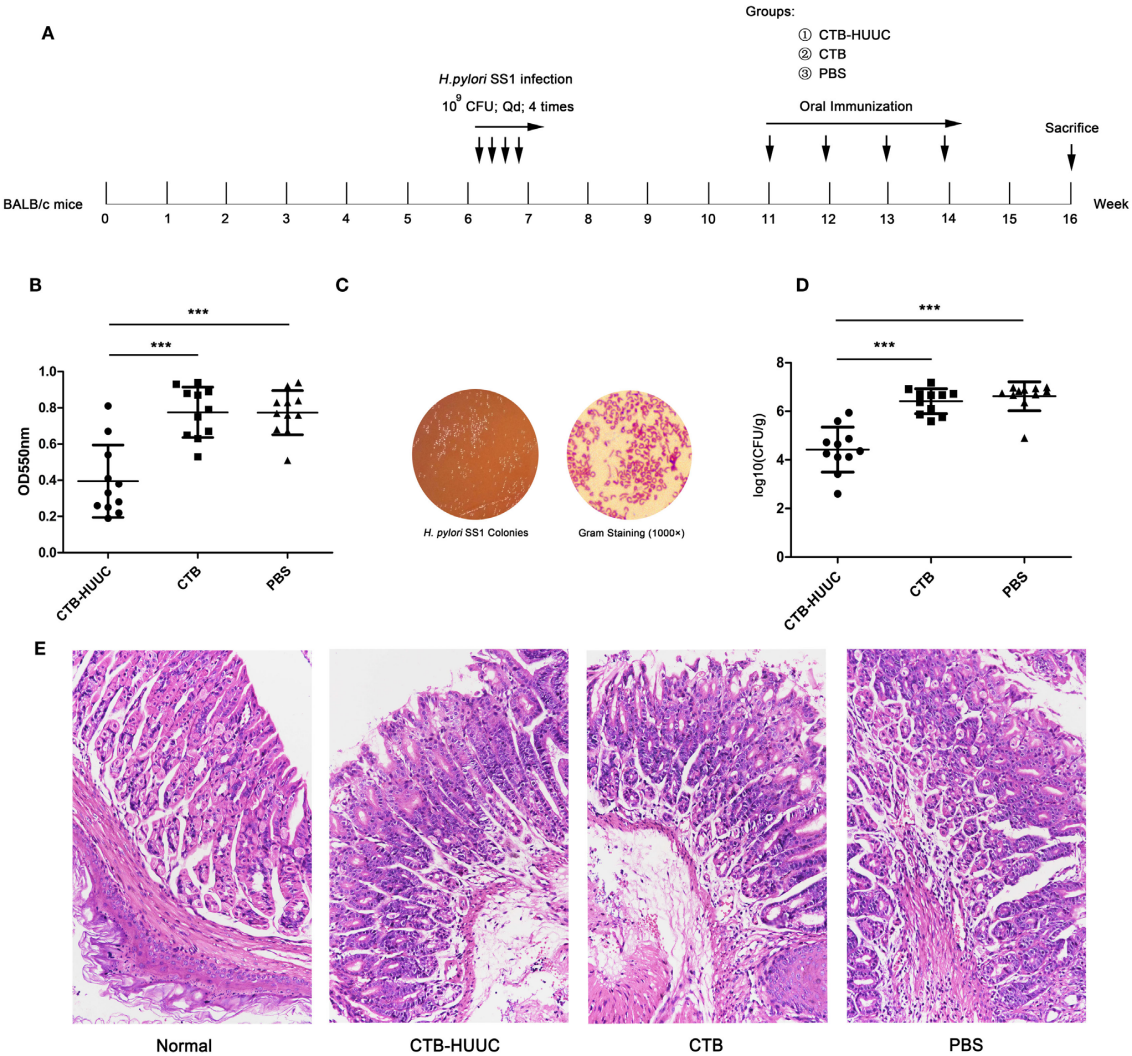
The effects of therapeutic vaccination with cholera toxin B subunit (CTB)-HUUC or CTB on *H. pylori* infection. **(A)** The therapeutic vaccination procedure. **(B)** The relative levels of urease activity in the stomach of mice immunized with oral administration of CTB-HUUC or CTB after *H. pylori* infection. ***p < 0.001, compared with the PBS group or between the CTB-HUUC and CTB groups. **(C)** Representative view of the *H. pylori* culture for colony formation assay. **(D)** The colony-forming units (CFUs) of *H. pylori* infection in the stomach of mice immunized with oral administration of CTB-HUUC or CTB after *H. pylori* infection. *H. pylori* infection was determined using quantitative culture. ***p < 0.001, compared with the PBS group or between the CTB-HUUC and CTB groups. **(E)** Gastric histological examination of mice immunized with oral administration of CTB-HUUC or CTB after *H. pylori* infection. The gastric tissue samples stained with hematoxylin and eosin were examined under a microscope (200×).

Therapeutic effect of the CTB-HUUC vaccine was also analyzed by bacterium quantitative culture, rapid urease test and gastric histological analysis. The results showed that therapeutic CTB-HUUC vaccination significantly decreased urease activity and reduced the *H. pylori* SS1 colonization in the stomachs, compared with CTB or PBS vaccination (*P* < 0.001) (Figures 6B–D). High levels of leukocytes and neutrophils were found in the stomachs from *H. pylori* SS1 infected BABL/c mouse model immunized with CTB or PBS. By contrast, inflammation was significantly weakened in the stomachs from *H. pylori* SS1 infected mice immunized with CTB-HUUC (Figure 6E).

## Discussion

Urease, HapA, CagA, and some other *H. pylori* proteins have been demonstrated to be excellent candidate antigens in animal models and even in human volunteers. A recent study reported that therapeutic immunization with a multivalent epitope-based vaccine (CFAdE) against four *H. pylori* adhesions (urease, Lpp20, HpaA, and CagL) could decrease the colonization of *H. pylori* by about four orders of magnitude (26). Interestingly, CFAdE, *H. pylori* lysate, and urease vaccinations induced comparable production of IFN-γ, IL-4, and IL-17, while CFAdE and *H. pylori* lysate had better protective efficacy compared with urease. The reason for this may be that oral vaccination with CFAdE and *H. pylori* lysate markedly elevated the level of serum IgG, stomach, and intestine mucosal sIgA against *H. pylori* compared with oral vaccination with urease. Zhou et al. (28) also showed that a multi-epitope vaccine LTB-HpaA-UreB (HUepi-LTB), containing three Th epitopes from UreB and two B cell epitopes from UreB and HpaA, oral therapeutic immunization with HUepi-LTB significantly decreased *H. pylori* colonization (about two orders of magnitude) compared with the PBS group, and the protection was correlated with mixed Th1–T-helper 2 (Th2) responses and IgG and mucosal IgA antibody responses. Given that the relationships between CagA and gastric cancer are well confirmed, an effective vaccine would be specifically targeting this toxin. Such a vaccine should be aimed at preventing *H. pylori*-induced serious illness rather than bacterial colonization (21). In a clinical trial (22), a multivalent vaccine, containing recombinant CagA, VacA, and NAP proteins, was immunogenic and safe. In our study, CagA149-164 and CagA196-217 were selected to construct the CTB-HUUC vaccine, and the results displayed that CagA in CTB-HUUC have good immunogenicity and immunoreactivity.

Despite vaccine efficacy against *H. pylori* infection has been shown in various animal models, the precise mechanisms of bacterial clearance remain relatively poorly understood (29, 30). A number of studies support the view that antibody production is not required to elicit immune protection, but some studies showed that humoral immune response is critical for clearing *H. pylori* (31, 32). Recently, an oral monovalent *H. pylori* vaccine using UreB fused with mucosal adjuvant heat-labile enterotoxin B subunit (LTA2B) has been found to be safe, immunogenic, and effective (71.8% protection rate) in *H. pylori* naive children aged between 6 and 15 years, in a randomized, double-blind, placebo-controlled, phase 3 clinical trial (33). Notably, they showed that 1-month serum anti-UreB IgG of 1:200 and salivary anti-UreB sIgA of 1:8 seemed to be optimum markers for a protection against *H. pylori* infection in volunteers. In addition, several previous studies have discovered that polyclonal antibodies produced by *H. pylori* urease immunization cannot inhibit urease enzymatic activity, whereas a number of monoclonal antibodies against *H. pylori* urease can (34–36). For example, L2 or HpU-2 monoclonal antibody recognized UreB327-334 or UreA183-203, respectively, and inhibited urease enzymatic activity (34, 36). Guo et al. (37) found that oral immunization with CTB–UreA_183–203_ (CTB–UA) could induce high levels of specific neutralizing antibodies which showed effectively inhibitory effect on the enzymatic activity of *H. pylori* urease, and significantly reduced *H. pylori* colonization in BABL/c mouse model. This group constructed another epitope vaccine named CTB–UreB_321–339_ (CtUBE), prophylactic or therapeutic vaccination with CtUBE significantly decreased *H. pylori* colonization, and the protection was correlated with antigen-specific IgG, IgA, and mucosal sIgA antibody responses (38). We speculated that humoral and local mucosal immune response might exhibit a certain protection against *H. pylori* infection, especially neutralizing antibodies against *H. pylori* urease, and the inhibition of bacterial adhesion may also contribute to clearance of the *H. pylori* infection. In our study, we formulated and constructed a multivalent vaccine named CTB-HUUC with three well know B-cell epitopes (HpaA132-141, UreA183-203, and UreB321-339) (Figure 1A). Oral vaccination with CTB-HUUC markedly elevated the level of serum IgG, stomach, and intestine mucosal sIgA against *H. pylori* compared with oral vaccination with CTB or PBS (Figures 2A,B), and purified mouse anti-(CTB-HUUC) IgG inhibited the urease activity in a dose-dependent manner (Figure 3E).

Whereas antibodies are dispensable for *H. pylori* protection, it is now clear that CD4^+^ T cells are critical for control of *H. pylori* infection (39). However, whether Th1, Th2, and T-helper 17 (Th17) polarized T cell subsets responses play dominant role in the protective immunity against *H. pylori* remains controversial. Earlier studies have demonstrated that Th2-cell responses are required for protective immunity against *H. pylori* infection, and Th1-cell responses are mainly involved in the pathogenesis of *H. pylori* (40, 41). However, some other studies showed that protective immunity against *H. pylori* involves specific CD4^+^ T cell Th1 type response (20, 42, 43). Meanwhile, a study reported that oral vaccination with HUepi-LTB significantly reduced *H. pylori* colonization in BABL/c mouse model, and the protection was correlated with a mixed Th1–Th2 phenotype (28). Moreover, some researchers have proposed that mixed Th1–Th17 cell responses are important for proper control of *H. pylori* infection (44, 45). In our opinion, the type of CD4^+^ T cell response may be controlled by choosing the corresponding type of antigen epitope. In this study, nine CD4^+^ T-cell epitopes (HpaA88-100, UreA27-53, UreB229-251, UreB317-329, UreB373-385, UreB438-452, UreB546-561, CagA149-164, and CagA196-217) were selected to construct the CTB-HUUC vaccine (Figure 1A). The lymphocyte proliferation results showed that splenic lymphocytes from mice immunized with CTB-HUUC proliferated significantly after stimulation with *H. pylori* lysate (Figure 4A). Furthermore, analysis of the cytokine production showed that IFN-γ (Th1 cells secrete), IL-4 (Th2 cells secrete), and IL-17 (Th17 cells secrete) were all significantly induced by CTB-HUUC (Figure 4B). Since CD4^+^ T cells were developed to Th1, Th2, and Th17 cells on the basis of their immune regulatory function and cytokine secretion profiles, Th1 cells predominantly produce IFN-γ and IL-2, Th2 cells secrete IL-4, IL-5, and IL-10, and Th17 cells secrete IL-17, IL-17F, and IL-22 (46). It is likely that the CTB-HUUC vaccine stimulated a mixed Th-cell response.

In conclusion, we produced a multivalent epitope-based vaccine CTB-HUUC with the intramucosal adjuvant CTB and tandem copies of B-cell epitopes and T-cell epitopes from HpaA, UreA, UreB, and CagA and assessed the efficacy of the CTB-HUUC in BALB/c mouse model. The results showed that both oral prophylactic and therapeutic CTB-HUUC vaccinations reduced bacterial load and protected stomachs in mice.

## Ethics Statement

This study was carried out in accordance with the recommendations of Animal Ethical and Experimental Committee of Hubei University of Medicine. The protocol was approved by the Animal Ethical and Experimental Committee of Hubei University of Medicine.

## Author Contributions

LP conceived the overall study and assisted in the design of experiments. XP, HK, and XN performed most experiments, assisted by SL and JL. LP, XP, SL and JL wrote the manuscript. All authors discussed the results and commented on the manuscript.

## Conflict of Interest Statement

The authors declare that the research was conducted in the absence of any commercial or financial relationships that could be construed as a potential conflict of interest.

## Footnotes

^1^http://tools.iedb.org/main/bcell/ (Accessed: June 22, 2017).

^2^http://tools.iedb.org/main/tcell/ (Accessed: June 22, 2017).
