# Supplementary material for: Protection Against Helicobacter pylori Infection in BALB/c Mouse Model by Oral Administration of Multivalent Epitope-Based Vaccine of Cholera Toxin B Subunit-HUUC
Source: Front Immunol. 2018 May 8;9:1003. doi: 10.3389/fimmu.2018.01003 (PMC5951970; doi:10.3389/fimmu.2018.01003)
Supplement: Supplementary file 1 [file Data_Sheet_1.docx]

**Table S1 Strains and plasmids used in this study**

| **Strains/plasmids** | **Features** | **Source** |
| --- | --- | --- |
| *Helicobacter pylori* SS1 | Mouse-adapted strain | ([Yang et al., 2015](#_ENREF_44)) |
| *Escherichia coli* BL21(DE3) | Host for protein expression | Tiangen Co., China |
| *Escherichia coli* Top10 | Host for gene cloning | Tiangen Co., China |
| pET28a(+) | Protein expression vector | TaKaRa Co., Ltd, China |
| pSUMO | Protein expression vector | TaKaRa Co., Ltd, China |
| pET28a(+)/*ctB-huuc* | Expression of CTB-HUUC | This study |
| pET28a(+)/*ctB* | Expression of CTB | This study |
| pSUMO/*hpaA* | Expression of HpaA | This study |
| pSUMO/*ureA* | Expression of UreA | This study |
| pET28a(+)/*ureB* | Expression of UreB | This study |
| pET28a(+)/*cagA* | Expression of CagA | This study |

**Table S2 Epitopes screened for the vaccine construction**

| **Epitopes** | **Features** | **Sequence** |
| --- | --- | --- |
| HpaA88-100 | CD4^+^ T-cell epitope | EQILQNQGYKVIS([Li et al., 2012](#_ENREF_23)) |
| HpaA132-141 | B-cell epitope | DPKRTIQKKS([Zhou et al., 2009](#_ENREF_46)) |
| UreA27-53 | CD4^+^ T-cell epitope | GVKLNYTEAVALISARVMEKARDGNKS([Guo et al., 2017a](#_ENREF_13)) |
| UreA183-203 | B-cell epitope | SVELIDIGGNRRIFGFNALVD([Guo et al., 2012](#_ENREF_11)) |
| UreB229-251 | CD4^+^ T-cell epitope | SAINHALDVADKYDVQVAIHTDT([Zhou et al., 2009](#_ENREF_46)) |
| UreB317-329 | CD4^+^ T-cell epitope | MLMVCHHLDKSIK([Li et al., 2012](#_ENREF_23)) |
| UreB321-339 | B-cell epitope | CHHLDKSIKEDVQFADSRI([Guo et al., 2013](#_ENREF_12)) |
| UreB373-385 | CD4^+^ T-cell epitope | ITRTWQTADKNKK([Li et al., 2012](#_ENREF_23)) |
| UreB438-452 | CD4^+^ T-cell epitope | SPAFFGVKPNMIIKG([Li et al., 2012](#_ENREF_23)) |
| UreB546-561 | CD4^+^ T-cell epitope | FVDGKEVTSKPANKVS([Zhou et al., 2009](#_ENREF_46)) |
| CagA149-164 | CD4^+^ T-cell epitope | NIIQPPIPDDKEKAEF([Li et al., 2012](#_ENREF_23)) |
| CagA196-217 | CD4^+^ T-cell epitope | KERQEAEKNGGPTGGDWLDIFL([Li et al., 2012](#_ENREF_23)) |
